# Heat treatment duration affects *in vitro*‐induced photosynthetic impairment and development of necrotic leaf tissue in three Mediterranean oak species

**DOI:** 10.1111/plb.70181

**Published:** 2026-01-18

**Authors:** N. Kunert, E. Düsterhöft, B. Stumpf

**Affiliations:** ^1^ Functional and Tropical Plant Ecology University of Bayreuth Bayreuth Germany

**Keywords:** Chlorophyll fluorescence, heat stress, necrotic tissue, stress duration

## Abstract

- Short heat treatments are commonly used to estimate the leaf‐level thermal tolerance of tree species. The effect of varying treatment duration has recently been challenged to have significant effects on the estimates of thermal tolerance thresholds and how it reflects the development of leaf necrosis. Here, we aim to elucidate the effect of treatment duration on the thermal tolerance estimates of three Mediterranean oak species.
- We applied heat treatments with varying treatment durations (15, 30, 60, 120 and 240 min) to excised leaves. The temperature response of the leaf maximum quantum yield of photosystem II (*F*_v_/*F*_m_) was measured immediately, 24 h, and 2 weeks after the treatment. Necrotic leaf damage was visually assessed 2 weeks after the treatment.
- Both the intensity of decline in *F*_v_/*F*_m_ and the development of necrotic tissue due to longer heat treatments were described by a logistic function. The longer the heat treatment lasted, the lower was the required temperature to induce irreversible damage to the photosystems and to reach a certain degree of visible damage to the leaf tissue. *F*_v_/*F*_m_ measurements carried out 24 h after the treatment predicted well the development of necrotic tissue.
- Leaf‐level thermal tolerance of Mediterranean oak species depends largely on the exposure duration to heat. The heat duration response follows a predictable logarithmic relationship, which allows for modelling the potential heat damage during heatwaves and forecasting climate change responses of Mediterranean forests.

## Graphical abstract

Longer lasting heat treatment durations induced photosynthetic impairment at lower temperatures and caused the development of proportionally more necrotic tissue at lower temperatures in leaves of three Mediterranean oak species.

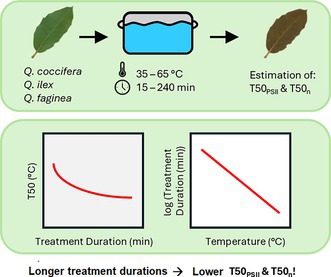

## INTRODUCTION

Abiotic forest disturbance has accelerated with climate change and in response higher tree mortality has been observed in almost every forest ecosystem worldwide (Allen *et al*. 2010; Cobb *et al*. 2017). The observed accelerating pace of tree mortality is significantly altering the composition of ecological communities, reducing ecosystem services by changing ecosystem functions and thereby leading to unpredictable land–climate feedback (Anderegg *et al*. 2013). Since the first record of climate‐change induced forest die‐off, it has become a major goal of forest research to shed light on the drivers and underlying mechanisms of tree mortality in order to reduce negative ecological, economic and cultural effects (Allen *et al*. 2010). Several possible drivers and mechanisms of tree mortality have been identified and are related to the water and carbon balances of trees. These drivers include physiological stress originating from drought, heatwaves, elevated atmospheric CO_2_ concentration and increasing atmospheric water vapour pressure deficit (Williams *et al*. 2013). Due to the co‐occurrence of these drivers and complex interdependencies of mechanisms, it remains complicated to decouple the actual contribution of each driver to the observed tree mortality (Hartmann *et al*. 2018; Hajek *et al*. 2020; Kunert 2020). Yet, species‐ and ecosystem‐specific information on drivers and mechanisms of tree mortality is crucial to take suitable mitigation actions.

In particular, negative effects of extreme temperatures during heatwaves have received increasing attention in temperate biomes lately (*e.g*., Kunert & Hajek 2023; Münchinger *et al*. 2023; Hauck *et al*. 2025). Various studies suggested that during summer heatwaves, many forest types already operate closely to their thermal tolerance thresholds (Tiwari *et al*. 2021; Kunert *et al*. 2022; Gauthey *et al*. 2024; Kullberg *et al*. 2024; da Porfırio Silva & Rossatto 2024). The estimates of thermal tolerance thresholds in these studies are all based on the assessment of the leaf‐level thermal tolerance. This is based on the common assumption that at the plant level, leaves are the most directly exposed organs to changes in ambient temperature at the atmospheric boundary layer and with this the most vulnerable organs (Atkin *et al*. 2006). Changes in temperature affect leaf photosynthesis and transpiration, which in turn are both fundamental processes determining plant performance (Way & Yamori 2014). Therefore, it is assumed that temperature‐dependent measures describing the leaf‐level photosynthetic decline will picture whole plant responses to heat stress (Wang *et al*. 2020).

Various standard protocols exist to assess the thermal tolerance thresholds of leaves *in vitro* based on temperature‐dependent changes in electrolyte leakage (*e.g*., Sullivan 1972), leaf chlorophyll fluorescence and development of necrosis (*e.g*., Bilger *et al*. 1984). Here, we want to focus on an established method to estimate thermal tolerance thresholds by exposing detached leaves to a fixed short‐term heat treatment in a water bath with a duration between 15 min (Krause *et al*. 2010; Tiwari *et al*. 2021; Winter *et al*. 2025) and 30 min (Larcher 1990; Münchinger *et al*. 2023; Neuner & Buchner 2023). These short‐term heat treatments are used to imitate the duration of heat peaks during the warmest time of the day (Sapper 1935; Larcher 1990). However, even significant differences in thermal tolerance thresholds within this narrow variation in time spectrum of 15–30 min have been described (Didion‐Gency *et al*. 2025). Even so, during climate change–induced heatwaves, the duration and exposure of leaves to high temperatures will be more frequent and intense (Perkins‐Kirkpatrick & Lewis 2020) and exceed the duration of short‐term temperature maxima (Neuner & Buchner 2023). Hence, the ecological relevance of such short‐term heat treatments has been questioned (Neuner & Buchner 2023). Further, Neuner & Buchner (2023) were able to show clear effects of duration on estimated temperature thresholds, with longer lasting treatments resulting in lower thermal tolerance. In conclusion, longer lasting treatments might be more appropriate to reflect heatwave scenarios (Neuner & Buchner 2023). Therefore, a comprehensive assessment of the temporal aspects regarding the duration of plant heat exposure is urgently needed to evaluate critical thresholds of thermal tolerance in the perspective of climate change.

The most common method of quantifying heat‐induced damage in leaves by the heat treatment is to measure the temperature‐dependent decline in maximum quantum use efficiency (*F*_v_/*F*_m_) of photosystem II (PSII) via chlorophyll fluorescence. PSII is characterized by a high heat sensitivity as it is a pigment–protein complex (Ashraf & Harris 2013) and when exposed to critical temperatures, irreversible damage occurs (Slot *et al*. 2021; Tiwari *et al*. 2021). Therefore, the temperature at which 50% of the maximum quantum use efficiency (T50_PSII_) is lost is considered a good indicator of irreversible damage (Krause *et al*. 2010; Tiwari *et al*. 2021). Recently, the representativeness of those temperature thresholds has been challenged to only poorly reflect the actual tissue damage in tropical tree species by the heat treatment (see Winter 2024; Winter *et al*. 2025). However, this relationship between T50_PSII_ and development of necrotic tissue has not been tested on trees growing in Mediterranean ecosystems.

In this study, we used the temperature‐induced changes in *F*_v_/*F*_m_ and estimated the post‐treatment development of necrotic tissue to assess thermal tolerance for three Mediterranean oak species. With this study, we want to (1) investigate the effect of the heat treatment duration on thermal tolerance thresholds, and (2) test the representativeness of *F*_v_/*F*_m_ values for the development of necrotic tissue damage. In addition, we wanted to provide an assessment of the thermal tolerance of three Mediterranean oak species.

## METHODS

### Study site and botanical material

**Fig. 1:**
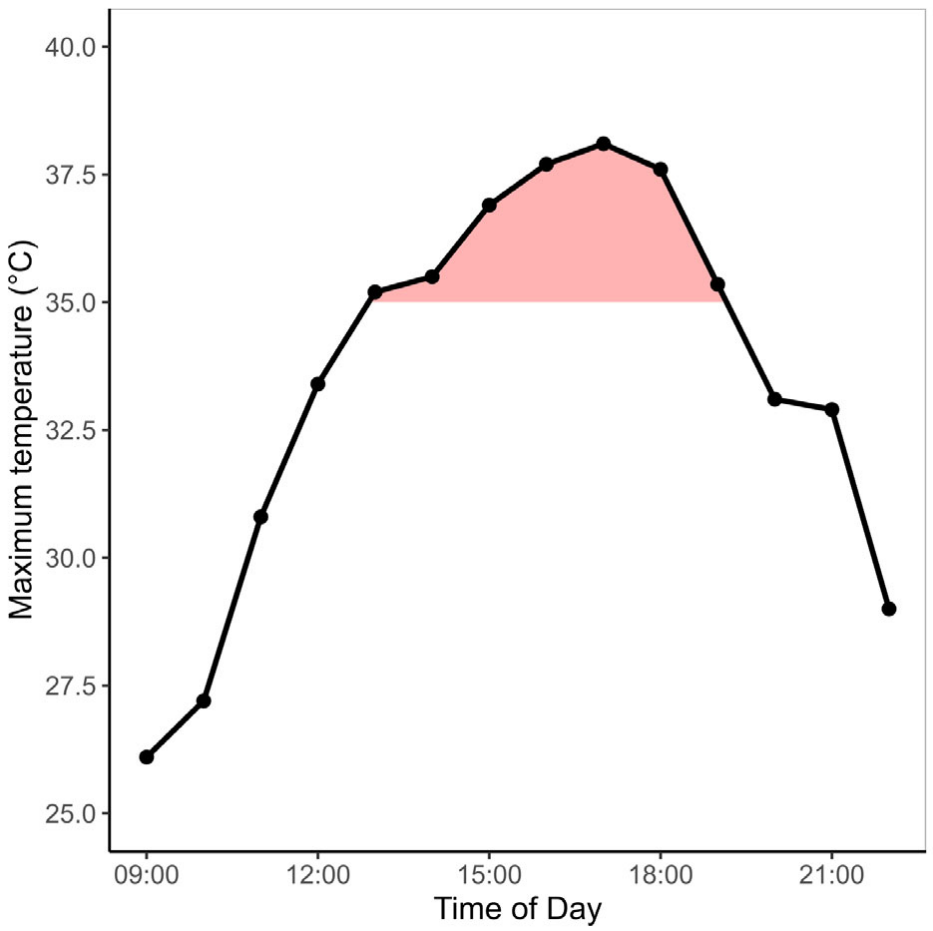
Diurnal course of the maximum temperature recorded on the hottest day on record (30 July 2024) at the closest climate station in Aínsa‐Sobrarbe (Ainsa la Serreta, station number: 9808X, AEMET 2025). The red‐shaded area indicates the heat duration above 35 °C on this day.

The botanical material for this study was collected on private property in the surroundings of the town of Olsón (42°16′59.12″ N 0°09′85.14″ E, 698 m a.s.l.) located in the municipality of Aínsa‐Sobrarbe, in the province of Huesca, Aragon, Spain. The area is characterized by a mild and moderate sub‐Mediterranean climate. The annual mean temperature at the nearby city of Aínsa‐Sobrarbe is 11.0 °C with an average amount of precipitation of 958 mm per year (AEMET 2025). The hottest day on record in the area was 30 July 2024, reaching a maximum temperature of 38.1 °C (Fig. 1). Temperatures above 37 °C lasted for 3 h on this specific day. The vegetation in the area is characterized by the typical meso‐xerophilous oakwoods of the pre‐Pyrenees. The oakwoods are open and are not forming a closed canopy. We focused on three oak species commonly found in the area, namely Portuguese oak (*Quercus faginea*), Holm oak (*Quercus ilex*) and Kermes oak (*Quercus coccifera*). The tallest trees are Portuguese oaks; however, they are mostly restricted to the lower elevations of 500–600 m a.s.l. Portuguese oak reaches heights below 20 m. Holm oak is the next tallest oak; its height declines with elevation. We found trees around 8 m in the lower parts of the geographical relief and trees with only 5 m in the higher parts. The oak with the lowest stature was Kermes oak. It dwells on the rocky outcrops where it is very abundant; however, it stays below a maximum height of 2 m. We collected botanical material for five mature individuals per species during the morning hours in September 2024. All individuals were fully exposed to the sun and not shaded by other trees. We cut one sun‐exposed branch per individual. As the stature of the trees is low and the canopy of the oakwoods remains open, we could easily reach the upper canopy with a 4‐m‐long pruner. Branches were placed in opaque plastic bags with moist tissue paper wrapped around the cuts and brought to the laboratory for further processing.

### Determination of heat damage

In the laboratory, we re‐cut the branches under water and placed them in buckets with water. Buckets were covered with opaque plastic bags and branches were left to re‐hydrate overnight. After re‐hydration, we chose healthy and undamaged leaves for the following measurements. All leaves were screened for their initial maximum photosynthetic efficiency (*F*_v_/*F*_m_) with a chlorophyll fluorometer (MINI‐PAM, Walz, Effeltrich, Germany). Therefore, the instrument was set to the ‘measuring light burst’ mode enabling an exact measurement of the initial fluorescence (*F*_o_). We used a low measuring light frequency of 0.6 kHz and a saturating light pulse of 1.0 s to record *F*_m_ (Münchinger *et al*. 2023). All leaves exhibited *F*_v_/*F*_m_ values between 0.75 and 0.83 (pre‐treatment measurements). In the following, we wanted to test the effect of varying temperature treatments, the effect of exposure duration on the vitality of the leaves, the recovery potential after the heat exposure and the development of necrotic leaf tissue. Therefore, we prepared 50 transparent sample sheets. On each sheet, we taped a set of leaves in the same order and each set consisted of 15 leaves (five per tree species and one leaf per sampled tree individual). We used air‐permeable adhesive tape (3M™, Micropore, Solventum Germany GmbH, Kamen, Germany) to attach the leaves to the sheets and placed damp paper towels on top of them. The sheets were double‐bagged in ziplock bags. Bags were randomly assigned to one of 10 temperature treatments; as we did treatments with five different treatment durations, there were five bags per temperature treatment. The temperature treatments were conducted in water baths at 35, 40, 42.5, 45, 47.5, 50, 52.5, 55, 60 and 65 °C. Temperature in the water baths was regulated with Sous‐Vide precision cookers. All bags were submerged in the water baths at the same time and one bag per temperature treatment was removed after 15, 30, 60, 120 and 240 min. Directly after the treatment, bags were opened and leaves dark adapted for at least 30 min and *F*_v_/*F*_m_ were re‐measured (in the following referred to as ‘immediate’ measurements). After those measurements, the leaves were left to recover at low light level for 24 h (Tiwari *et al*. 2021) and *F*_v_/*F*_m_ was re‐measured (in the following referred to as ‘24 h’ post‐treatment measurements) following the same protocol. We expected a recovery of *F*_v_/*F*_m_ between the measurements immediately after the treatment and after a 24‐h recovery period (Krause *et al*. 2010). To test for permanent damage, *F*_v_/*F*_m_ was re‐measured after 2 weeks in order to compare it with the development of necrotic tissue (Winter *et al*. 2025). Therefore, leaves were left at room temperature in the ziplock bags, under low light level and the paper tissue was re‐wetted if necessary. We measured *F*_v_/*F*_m_ after 2 weeks again, following the exact same protocol as mentioned above (in the following referred to as the ‘2 weeks’ measurement). In addition to *F*_v_/*F*_m_ measurements, we visually assessed the percentage of brown necrotic area relative to the total leaf area of all leaves after 14 days (Kunert & Gebhard, 2026). The estimation of the necrotic area was done independently from the chlorophyll fluorescence measurements. To test if the first temperature treatment at 35 °C had any effect on the measurements, we compared the pre‐treatment measurements with the post‐treatment measurements using a paired samples *t*‐test. Mean pre‐treatment and post‐treatment values are presented with ± SD. The *t*‐test was performed in JASP, version 0.19.0 (JASP Team 2024). We applied a linear mixed‐effects model to evaluate the variance of the relationship between T50_PSII_ and T50_n_ by random effects (package ‘lme4’ Bates *et al*. 2015 and ‘lmerTest’ Kuznetsova *et al*. 2015). Therefore, we used T50_n_ as the dependent variable, T50_PSII_ as the fixed effect, species and treatment duration as random factors.

### Estimation of temperature thresholds and decline velocities

We used the approach by Buchner *et al*. (2013) to estimate the effect of the heat treatment on chlorophyll fluorescence. In this approach, the relative decrease of the quantum use efficiency of the PS II (Δ*F*_v_/*F*_m_ in %) is calculated as follows:

(1) $$\Delta F_{v}/F_{m}=100\;\frac{{\left(F_{v}/F_{m}\right)}_{\mathrm{ref}}-{\left(F_{v}/F_{m}\right)}_{\text{heat}}}{{\left(F_{v}/F_{m}\right)}_{\mathrm{ref}}}$$

where *F*_v_/*F*_m_ is measured for each leaf before (*F*_v_/*F*_m_)_ref_ and after the temperature treatment (*F*_v_/*F*_m_)_heat_. The response to the temperature treatments was determined in the following using a log‐logistic curve as described by Kunert *et al*. (2022):

(2) $$\Delta F_{v}/F_{m}=c+\frac{d-c}{1+\mathrm{Exp}\left[b\;\log\left(\frac{T}{T50_{\text{PSII}}}\;\right)\right]}$$

where *T* describes the temperature, *c* the Δ*F*_v_/*F*_m_ of the lower plateau and *d* the higher plateau. T50_PSII_ represents the turning point of the function and the temperature at which Δ*F*_v_/*F*_m_ decreases by 50%. Parameter *b* represents the slope of the curve at T = T50_PSII_. The response curves were fitted using the ‘modelFit’ function of the ‘drc’ package (Ritz *et al*. 2016) in R. From the curve, we extracted the turning point of the function that reflects T50_PSII_, which is the temperature‐dependent decline of the photosynthetic quantum use efficiency by 50%. The presented means of T50_PSII_ are with ± SE. We fitted a simpler model to treatments and species pairs to identify differences in T50_PSII_ between treatments and species. Therefore, we compared the deviations from the treatment‐ and species‐specific dose–response curves with an approximate *F*‐test and the ‘compParm’ in case that the null hypothesis was rejected (Ritz *et al*. 2016).

We evaluated the effect of treatment duration on the decline of *F*_v_/*F*_m_ (T50_PSII_) and the development of necrotic tissue (T50_n_) using the approach by Neuner & Buchner (2023). In the equations, T50 will be used instead of T50_PSII_ and T50_n_ as we used the same procedure to determine the relationship between these estimates and the treatment duration. First, we applied a logarithmic function:

(3) $$T50=k\ln\left(t\right)+d$$

where the parameter k is a coefficient describing the slope. The parameter t is the exposure duration and d is a constant. In the next step, we calculated the function that described the species‐specific exposure duration that leads to a given leaf damage. This was done by inverting the functions from equation 2. Therefore, the function is given as an exponential function:

(4) $$t=\exp\left(\frac{T50-d}{k}\right)$$

The velocity of the treatment‐dependent T50 can be given as the first derivative (T50) of the logarithmic function 3 and it becomes possible to calculate

(5) $$T50^{\prime }=k\;t^{-1}$$

The velocity is given as dT50/dt in Kh^−1^. dT50/dt describes the change in the measured T50 value with a longer lasting treatment duration (Neuner & Buchner 2023). Data analysis was performed using the R program, version 4.4.3 (R Core Team 2025).

## RESULTS

### Thermal tolerance of Mediterranean oaks

Mean T50_PSII_ across the three oak species after a standard 30‐min heat treatment and measured after 24 h was 48.9 ± 1.7 °C (Table 1). Kermes oak was the species with the highest thermal tolerance and T50_PSII_ was at 50.1 ± 0.4 °C. The second highest T50_PSII_ of the three oak species was found in holm oak (49.7 ± 0.7 °C). Portuguese oak had the lowest T50_PSII_ (46.9 ± 0.8 °C) measured under the described standard conditions.

**Table 1:** Summary of the measured thermal tolerance for the three Mediterranean oak species measured immediately after the treatment, after a 24‐h recovery period after the heat treatment and 2 weeks after the treatment.

common name	Kermes oak			Portuguese oak			Holm oak		
Latin name	*Quercus coccifera*			*Quercus faginea*			*Quercus ilex*		
treatment duration	T50_PSII_	T50_PSII_	T50_PSII_	T50_PSII_	T50_PSII_	T50_PSII_	T50_PSII_	T50_PSII_	T50_PSII_
minutes	°C ± SE	°C ± SE	°C ± SE	°C ± SE	°C ± SE	°C ± SE	°C ± SE	°C ± SE	°C ± SE
15	50.9 ± 1.0^a,A^	49.2 ± 0.7^b,A^	51.3 ± 0.4^a,A^	47.7 ± 0.6^a,B^	46.6 ± 0.3^a,A^	NA	54.9 ± 0.6^a,C^	52.4 ± 0.7^b,B^	51.7 ± 0.2^c,A^
30	50.4 ± 0.5^a,A^	50.1 ± 0.4^a,A^	49.7 ± 0.2^b,A^	44.1 ± 0.5^a,B^	46.9 ± 0.8^b,B^	NA	52.4 ± 0.8^a,A^	49.7 ± 0.7^b,A^	50.3 ± 0.3^b,A^
60	46.7 ± 0.8^a,A^	46.5 ± 0.8^a,A^	52.5 ± 0.9^b,A^	42.8 ± 1.1^a,A^	45.5 ± 0.9^a,B^	NA	49.4 ± 0.4^a,C^	47.4 ± 0.5^b,A^	48.9 ± 0.3^b,B^
120	44.3 ± 0.5^a,A^	45.2 ± 0.4^a,A^	46.5 ± 0.3^b,A^	42.8 ± 0.4^a,B^	43.4 ± 0.2^b,B^	NA	45.6 ± 0.3^a,C^	46.0 ± 0.3^a,A^	46.0 ± 0.2^a,A^
240	44.8 ± 0.5^a,A^	44.8 ± 0.5^a,A^	44.5 ± 0.9^a,A^	44.5 ± 0.8^a,A^	44.8 ± 0.4^a,A^	NA	45.2 ± 0.3^a,A^	44.8 ± 0.4^a,A^	44.8 ± 0.3^a,A^
Time of measurements	Immediate	24 h	2 weeks	Immediate	24 h	2 weeks	Immediate	24 h	2 weeks

Values reflect the temperature at which the PSII efficiency declines by 50% (T50_PSII_) of the maximum. Small letters indicate significant differences between the time of measurements within a species. Capital letters indicate significant differences between species within a time of measurement.

### Effect of heat dose on thermal tolerance values

**Fig. 2:**
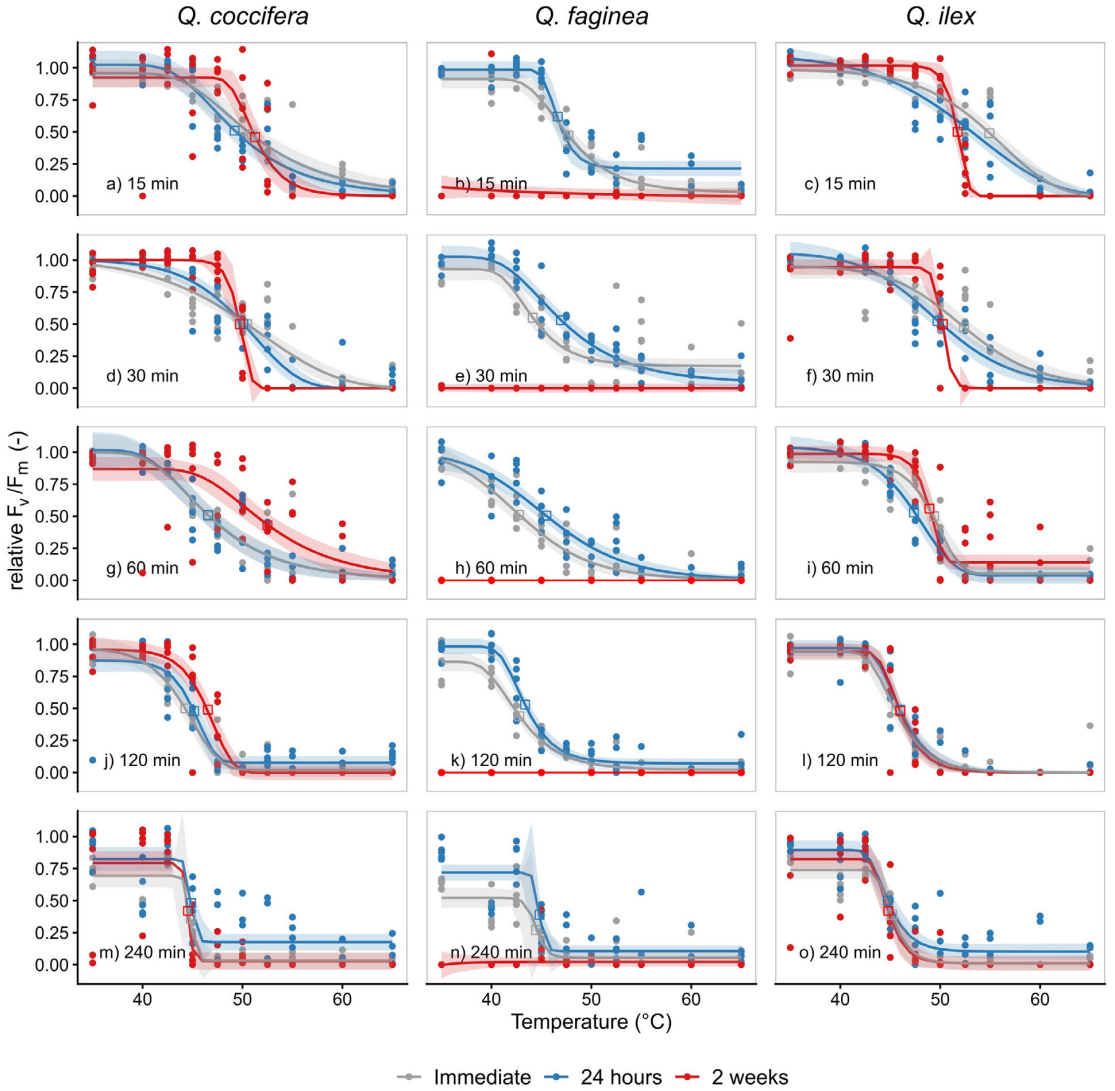
Temperature response of PSII efficiency (*F*_v_/*F*_m_) of the three oak species (a, d, g, j, m: *Quercus coccifera*; b, e, h, k n: *Quercus fagina*; c, f, i, l, o: *Quercus ilex*) to different treatment durations assessed immediately after the heat treatment, 24 h after and 2 weeks after the treatments for the three oak species. Five individuals per species and treatment were measured to establish the thermal vulnerability curve. The coloured bands represent the 95% confidence intervals.

**Fig. 3:**
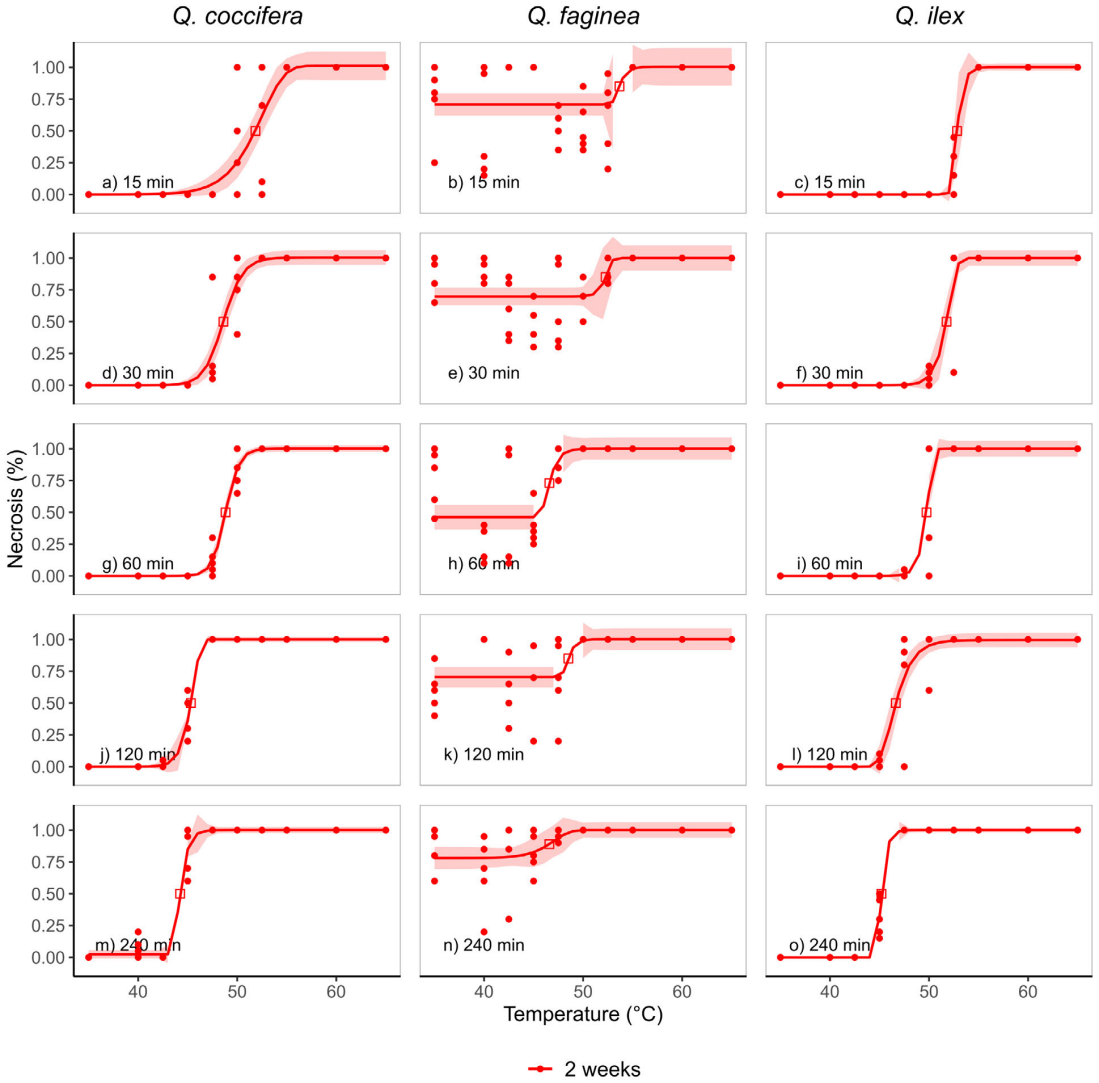
Leaf necrosis in response to different treatments and treatment durations estimated 2 weeks after the treatments for the three oak species (a, d, g j, m: *Quercus coccifera*; b, e, h, k, n: *Quercus fagina*; c, f, i, l, o: *Quercus ilex*). Five individuals per species and treatment were measured to establish the thermal vulnerability curve. The coloured bands represent the 95% confidence intervals.

There were clear patterns of the effect of increasing temperatures on the decline of *F*_v_/*F*_m_ (Fig. 2) and the development of necrosis (Fig. 3). All temperature‐dependent *F*_v_/*F*_m_ measurements followed clear dose–response curves (Fig. 3), regardless of the time when the chlorophyll fluorescence measurements were conducted. However, the leaves of Portuguese oak started to disintegrate rapidly, and all leaf samples had a *F*_v_/*F*_m_ of 0 after 2 weeks (Fig. 2). The relationship between heat treatment and the development of necrotic leaf tissue could also be described by dose–response curves (Fig. 3).

We observed a significant difference in the *F*_v_/*F*_m_ values between the pre‐treatment measurements (mean = 0.783, SD = 0.028) and the post‐treatment measurements (mean = 0.721, SD = 0.076) (t(14) = 3.958, *P* = 0.001) at 35 °C and treatment duration of 240 min among all species. In the other treatment duration, there was no significant difference between the pre‐treatment measurements and the post‐treatment measurements (all treatments *P* > 0.068). There was no effect of the 15‐min heat treatment on the *F*_v_/*F*_m_ values compared to the pre‐treatment measurements (mean = 0.754, SD = 0.022) even if a slight but not significant increase from post‐treatment measurements (mean = 0.771, SD = 0.031) (t(14) = −1.810, *P* = 0.092) was measurable.

**Fig. 4:**
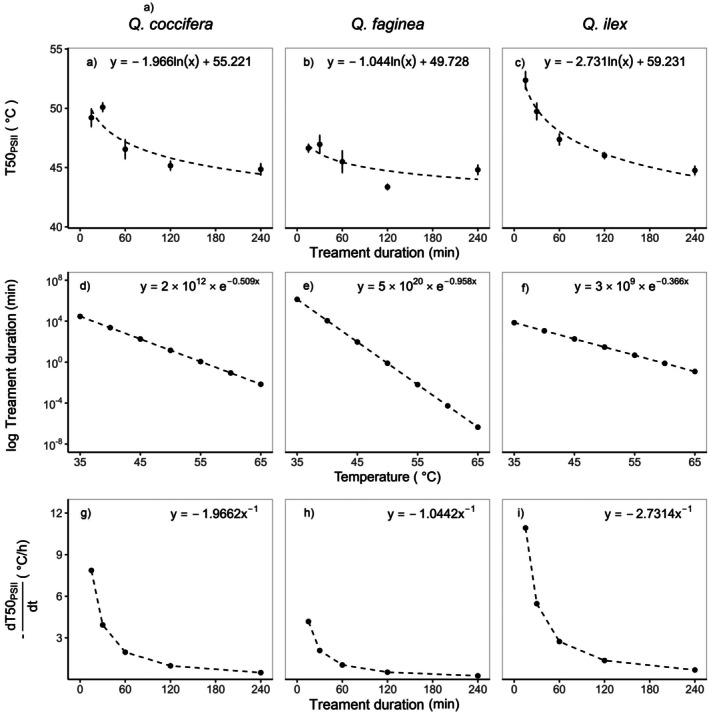
Effect of treatment duration on thermal tolerance thresholds expressed as the temperature at which the quantum use efficiency in the PSII (*F*_v_/*F*_m_) declines by 50% (T50_PSII_) for the three oak species. The three panels on the top (a–c) show the relationship between T50_PSII_ values and the treatment duration. The three panels in the middle (d–f) show the inverted logarithmic function. The inverted function enables the estimation of the heat treatment duration that causes T50 to decline as a function of temperature (compare Neuner & Buchner 2023). The three panels on the bottom (g–i) present the effect of the treatment duration on dT50/dt (±SD) for the three oak species. dT50/dt [K·h^−1^] is the current rate of change in T50_PSII_ determined at a given treatment duration.

**Fig. 5:**
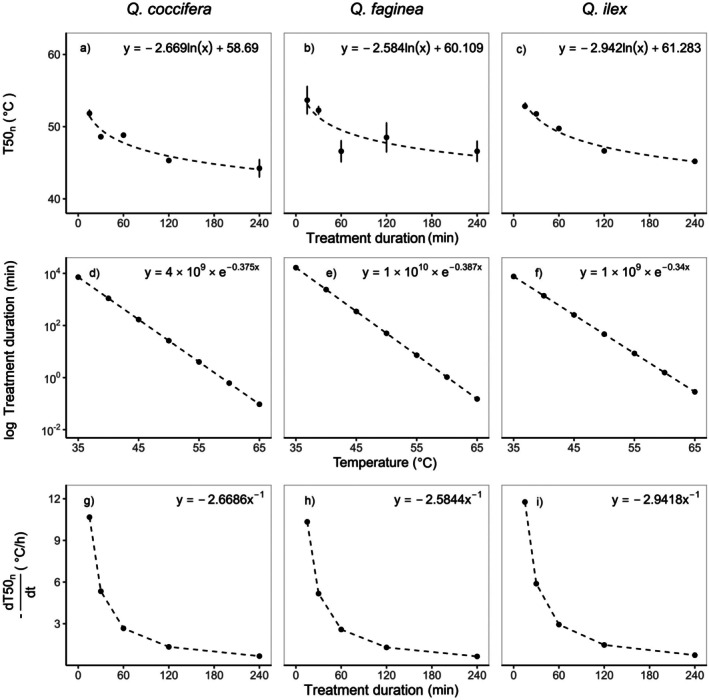
Effect of treatment duration on thermal tolerance thresholds estimated from the development of necrotic tissue after 2 weeks. The values represent the temperature when 50% of the tissue is visibly dead (T50_n_). The three panels on the top (a–c) show the relationship between T50_n_ values and the treatment duration. The three panels in the middle (d–f) show the inverted logarithmic function. The three panels on the bottom (g–i) present the effect of the treatment duration on dT50/dt (±SD) for the three oak species. dT50/dt [K·h^−1^] is the current rate of change in T50_n_ determined at a given treatment duration.

T50_PSII_ of all species declined rapidly with increasing treatment duration (Fig. 4); the same was true for the development of necrotic tissue (T50_n_ Fig. 5). The decline of T50 values (T50_PSII_ and T50_n_) in response to increasing treatment duration followed a classic logistic response. There was a high deviation of T50_PSII_ derived from shorter and longer treatment durations. These T50_PSII_ were either lower or higher relative to T50_PSII_ calculated from the standard treatment duration of 30 min. For example, a treatment duration of 15 min resulted in a T50_PSII_ of 49.2 ± 0.7 °C in Kermes oak and 44.8 ± 0.5 °C after a 240‐min lasting treatment (*F*_v_/*F*_m_ measured after 24 h, Table 1). Curiously, all three species had the same T50_PSII_ of 44.8 °C after a 240‐min‐long heat treatment. Overall, the difference between the 15 and 240 min treatments ranged between 3.2 and 9.7 °C, regardless of species and the time when *F*_v_/*F*_m_ was measured.

T50_PSII_ values were significantly different in some species when measured 24 h after the treatment compared to the immediate measurements after the treatment and 2 weeks after the treatment (Table 1). However, leaves of Portuguese oak degraded rapidly, and *F*_v_/*F*_m_ were all around zero after a 2‐week post‐culture. The differences were indicated between the timing of measurements (Table 1), and those differences were only present in the short treatment duration and vanished the longer the heat treatments lasted. For example, all T50_PSII_ values measured immediately after the treatment were significantly different to the T50_PSII_ values measured 24 h after a 30‐min‐long heat treatment in all three oak species. T50_PSII_ values measured after a 240‐min‐long heat treatment duration did not show any difference when assessed immediately, 24 h or 2 weeks after the treatment. Even species differences in T50_PSII_ disappeared after this long treatment duration (Table 1).

*F*_v_/*F*_m_ measurements were characterized by a fast change in dT50/dt at treatment durations of 15 and 30 min (Fig. 4) and the same applies for the T50_n_ values (Fig. 5). We need to note that in Kermes and Holm oak, T50_PSII_ values were lower after a 15‐min treatment than after a 30‐min treatment, whereas all T50_n_ values estimated after a 15‐min treatment were higher than after a 30‐min treatment. However, the estimated dT50/dt at 15 min from T50_PSII_ was at −4.2 Kh^−1^ in Portuguese oak, −8.0 Kh^−1^ in Kermes oak and −10.9 Kh^−1^ in Holm oak. A dT50/dt of −1.0 Kh^−1^ was reached after a treatment duration of 63 min in Portuguese oak, 120 min in Kermes oak and 164 min in Holm oak. dT50/dt derived from the estimation of the necrotic tissue development was much faster with lower differences between the three species. For example, at a 15‐min treatment duration, dT50/dt was −10.3 Kh^−1^, −10.7 Kh^−1^ and −11.8 Kh^−1^ in Portuguese oak, Kermes oak and Holm oak, respectively. A dT50/dt of −1.0 Kh^−1^ was reached after 150 , 159 and 170 min in Portuguese oak, Kermes oak and Holm oak, respectively.

### Chlorophyll fluorescence versus necrotic tissue

**Fig. 6:**
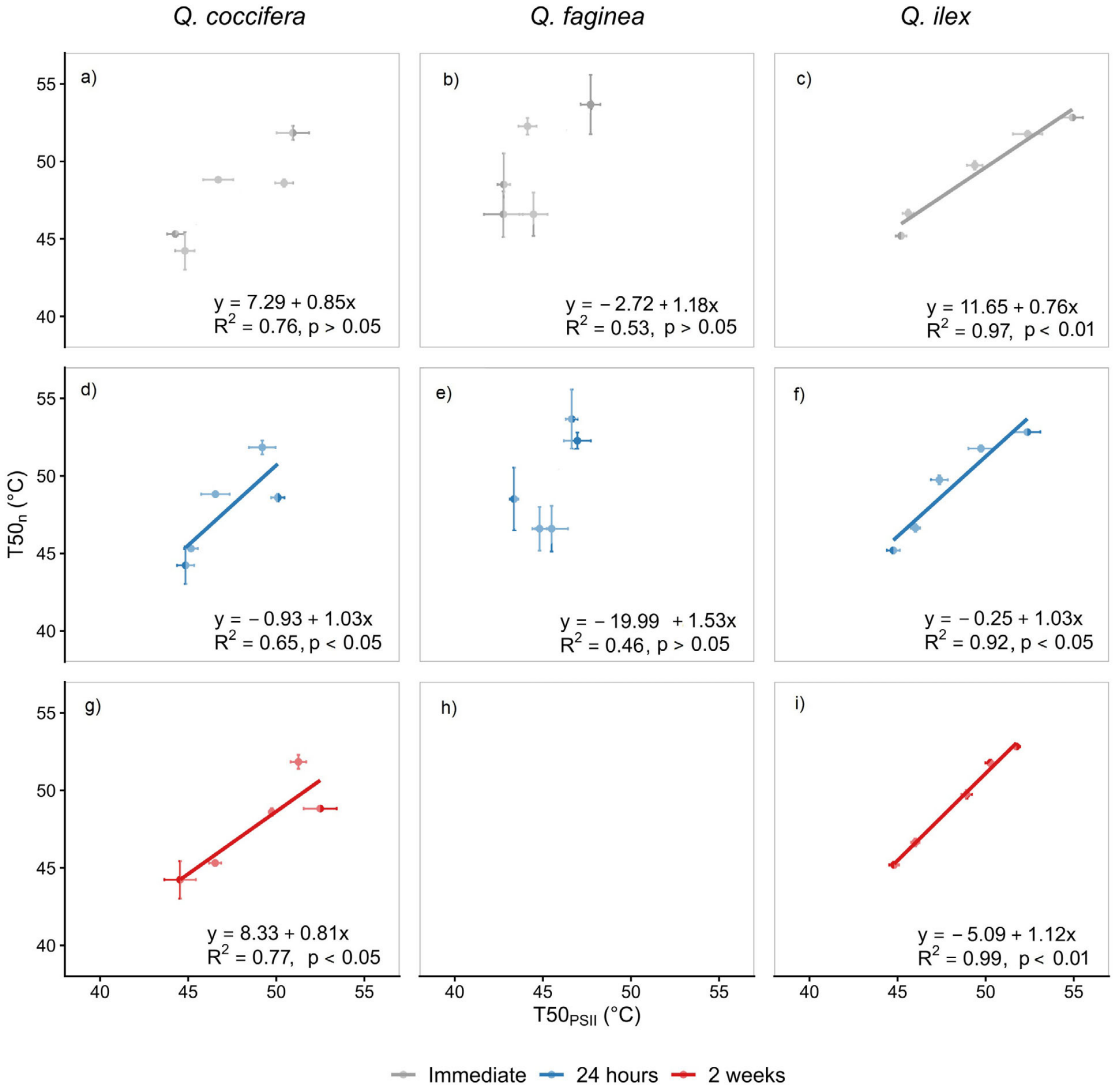
Linear relationship between thermal tolerance thresholds estimated via chlorophyll fluorescence (T50_PSII_) assessed immediately after the treatment (a–c), 24 h (d–f) and 2 weeks (g–i) after the treatment plotted against the visibly assessed of necrotic tissue development (T50_n_) after 2 weeks post‐culture. Error bars indicate the standard error. T50_PSII_ could not be estimated for Portuguese oak (*Quercus faginea*) after 2 weeks after the treatment; therefore, panel (h) is left empty.

All T50_PSII_ values, regardless of the time of measurement and despite that chlorophyll fluorescence measurements could not be repeated in Portuguese oak after 2 weeks, reflected well the development of necrotic tissue (T50_n_, Table 2 and Fig. 6). However, in both Kermes oaks and Holm oaks, the relationship between T50_n_ and T50_PSII_ was described by a slope of 1.03 in the chlorophyll fluorescence measurements after 24 h, indicating a good agreement between T50 derived from the two different estimates. Portuguese oak has a slope of 1.53 comparing the necrotic tissue with the chlorophyll fluorescence measurements after 24 h. Chlorophyll fluorescence measurements immediately conducted after the treatments had lower slopes in Kermes and Holm oak (0.85 and 0.76, respectively). The relationship between chlorophyll fluorescence measurements in Kermes oaks and Holm oak conducted after 2 weeks did not show similar patterns in the two species, as the slope in Kermes oak was below 1 and in Holm oak above 1. Treatment duration explained 85.5% of the total variance of the relationship and species 10.7% of the total variance of the relationship.

**Table 2:** Summary of the estimated necrosis for the three Mediterranean oak species visually assessed 2 weeks after the treatment.

common name	Kermes oak	Portuguese oak	Holm oak
Latin name	*Quercus coccifera*	*Quercus faginea*	*Quercus ilex*
treatment duration	T50_n_	T50_n_	T50_n_
minutes	°C ± SE	°C ± SE	°C ± SE
15	51.8 ± 0.5^A^	53.7 ± 1.9^A^	52.8 ± 0.2^A^
30	48.6 ± 0.2^A^	52.3 ± 0.5^A^	51.8 ± 0.2^B^
60	48.8 ± 0.1^A^	46.6 ± 1.4^A^	49.7 ± 0.3^A^
120	45.3 ± 0.2^A^	48.5 ± 2.0^A^	46.6 ± 0.2^B^
240	44.2 ± 1.2^A^	46.6 ± 1.4^A^	45.2 ± 0.2^B^

Values reflect the temperature at which 50% (T50_n_) of the leaf area developed necrotic tissue. Capital letters indicate significant differences between species within a treatment duration.

## DISCUSSION

Increasing treatment duration had a significant effect on the photosynthetic quantum use efficiency in all three Mediterranean oak species. The same effect could be observed in the development of necrotic tissue 2 weeks after the treatment. The longer the heat treatment lasted, the lower the required temperature was to induce irreversible damage to the PSII and to cause visible necrotic damage to the leaves. The relationship between the duration of heat treatments and the thermal tolerance values is described by a logarithmic function. We found that species differences in the estimated temperature thresholds disappear with treatments lasting 240 min. The best agreement between the heat‐induced damage to the PSII and the visible necrotic damage was found for the chlorophyll fluorescence measurements 24 h after the heat treatment.

### Influence of heat duration

Our study shows that the duration of the heat treatment affects the intensity of the damage caused to the PSII. Prolonging the heat treatment from 15 to 240 min lowered the T50_PSII_ values between 1.8 and 7.6 °C after a 24‐h recovery period (Portuguese oak and holm oak, respectively). However, the reduction in T50_PSII_ values by varying treatment duration can diverge even more when the span between the treatment durations is wider. For example, Neuner & Buchner (2023) exposed leaves of alpine herbs, shrubs and trees to heat treatments between 1 and 512 min and could induce a lowering of the T50_PSII_ values by more than 10 °C in alpine plant species. Hence, the duration of thermal stress during a heatwave does crucially influence the consecutive damage. High thermal stress lasting longer than 30 min does induce significantly higher damage. Unfortunately, there is, to the best of our knowledge, no comprehensive analysis of how long high temperatures last during a typical day of a heatwave. However, studies on tree thermal tolerance usually assume that any reached maximum air temperatures negatively affect plant physiological processes, independent of their duration (*e.g*., Tiwari *et al*. 2021; Kunert *et al*. 2022). The actual duration of the maximum temperatures is not considered in those observations. The only study we know that directly compares daily maximum temperatures and their duration with thermal thresholds (T5: 42.2 °C; T50: 48.6 °C; and T95: 52.7 °C) found that air temperatures surpassed locally 40 °C for 3.8 h during a heatwave on one hot day (Kunert 2024). Higher temperatures such as 41 °C were reached and surpassed for 90 min. The same study reports that an air temperature above 42 °C lasted for 40 min. Those air temperatures and their duration are close to the common assumption of what happens during a heatwave. For example, in a manipulative heat experiment, Cox *et al*. (2025) used a treatment duration of 2 h to simulate a heat wave with a reasonable peak temperature of 41 °C. A study investigating leaf temperature in an alpine dwarf shrub during a regular summer showed that high leaf temperatures above approximately 40° C can persist for 4.4 h (Neuner & Buchner 2023). However, leaf temperatures of the Austrian oak (*Quercus cerris*) in the study by Kunert (2024) never reached temperatures as high as the ambient air temperatures. Taller trees seem to have the ability to cool down the leaves effectively, whereas small plants can excessively exceed temperatures compared to air temperature (Körner 2016). For example, in the generally smaller statured Holm and Kermes oak canopy, temperature exceeds air temperature during the daytime (Gauthey *et al*. 2024), indicating the need of higher thermal tolerance properties or protective mechanisms such as leaf cooling. In particular, leaf cooling mechanisms may help prevent excessive heat damage, potentially extending the leaf's thermal safety margin (Gauthey *et al*. 2024).

Despite an initial significant difference between the three species in their T50_PSII_ values after the exposure to 15 min of heat and a recovery period of 24 h, this difference vanished, and all three oak species had the same T50_PSII_ values of 44.8 ± 0.2 °C after an exposure to heat of 240 min. Curiously, this was independent from the time when measurements were carried out. Thus, after being exposed to high temperatures for various hours, all species seem to equalize in their sensitivity to heat stress. In terms of climate‐change‐induced heatwaves, long‐lasting heat episodes could affect different species that are currently thought to be characterized by different thermal tolerance thresholds equally. This observed decline in T50 values (both T50_PSII_ and T50_n_ values) in dependency of treatment duration followed a logarithmic relationship. Regarding T50_PSII_, dT50/dt slowed down to −1 Kh^−1^ when treatments lasted between 1 and 2 h, whereas the Portuguese oak did reach −1 Kh^−1^ faster than the other two species. Portuguese oak is characterized by softer leaves than Kermes and Holm oak. The latter two species have approximately twice the leaf mass per area (LMA) than Portuguese oak (Mediavilla & Escudero 2003; Alonso‐Forn *et al*. 2023). Hence, T50 values themselves (Münchinger *et al*. 2023) and the behaviour of how T50 changes with treatment duration is linked to other species‐specific morphological traits such as LMA. An important aim of the estimation of single threshold temperatures is to reflect such species‐specific traits. As species differences disappear with increasing treatment durations longer than 1 or 2 h, we conclude that it is not advisable to exceed the commonly used treatment durations of 15 or 30 min.

### Necrosis as estimate for tissue damage

Our results show that *F*_v_/*F*_m_, independently when it was measured, represents the post‐heat treatment development of irreversible damage in leaf tissue. However, we found the best agreement, a slope of 1, after a recovery period of 24 h, in particular after 15‐ and 30‐min heat treatment duration. This is in accordance with the protocol established by Krause *et al*. (2010) who described a recovery of *F*_v_/*F*_m_ after a 24‐h recovery period in *Ficus insipida* after initial 15‐min lasting heat treatments. The T50_PSI_ values were further well correlated with T50_n_, suggesting that a 24‐h recovery period after the treatment is adequate. This contrasts with findings by Winter *et al*. (2025) who could not confirm this relationship between the temperature‐dependent decline in *F*_v_/*F*_m_ and necrotic tissue as there was significant tissue recovery during the post‐treatment period. On the other hand, we found that the suitability of leaf samples for a post‐treatment culture depends largely on the leaf structure. The two species with good agreement, again a slope of 1, between T50_PSII_ measured after 24 h and T50_n_ were Kermes oak and Holm oak. Both species have thick and tough leaves, as mentioned above. In these two species, a low necrosis could be identified in the lower treatment temperatures after 2 weeks post‐culture. Tissue was visibly alive, and leaves were still characterized by high *F*_v_/*F*_m_ values (~0.80). On the other side, Portuguese oak has a low LMA, thus much thinner leaves. The thin leaves of Portuguese oak started to degrade rapidly after the treatment and a high percentage of necrosis was present after a 2‐week‐long post‐culture, even at low temperature treatments (*e.g*., ~75% necrosis at 35 °C). This confirms the findings by Winter *et al*. (2025) who suggested that leaf characteristics might have a crucial influence on the T50_PSII_ and T50_n_ relationships and that this relationship needs to be confirmed for a much larger variety of tree species in different biomes. Further, we agree with Winter *et al*. (2025) that there is an urgent need for experiments exposing whole plants to heat to further improve our understanding of how intense heat stress affects the long‐term development of plants.

### Thermal tolerance following the 30‐min standard protocol

The differences in the thermal tolerances of the three oak species reflected the adaptation to certain habitats in the sub‐Mediterranean and Mediterranean landscapes. The two species, namely Kermes oak and Holm oak, showed a high thermal tolerance, whereas Portuguese oak was characterized by a lower thermal tolerance. The first two species are known to be well adapted to arid sites with Kermes oak occupying habitats in the climatic extremes regarding aridity (Braun‐Blanquet & Bolos de 1957). Holm oak is known for its effective photoprotective mechanisms enabling a high drought resistance (Alonso‐Forn *et al*. 2021). In contrast to Kermes and Holm oak, Portuguese oak requires cooler and deeper soils with more moisture (Castro‐Díez *et al*. 1997) and appears to be less resistant to prolonged summer drought and an increase in the soil water recharge favours the growth of this species (Kouba *et al*. 2011). Besides the differences in habitat requirements, the higher thermal tolerance is also manifested in the expression of leaf functional and physiological traits other than the measured T50 and underpins the value of the short duration treatments. For example, thicker leaves (higher LMA) and leaves shrinking less upon desiccation (lower percentage loss of area, PLA) represent a higher investment in leaf structure resulting in higher leaf rigidity and xeromorphy (Münchinger *et al*. 2023). Accordingly, leaves of Kermes and Holm oak (PLA: 7.9% and 9.3%, respectively; unpublished data, measured in the Ecological‐Botanical garden in Bayreuth) shrink less upon desiccation than leaves of Portuguese oak (PLA: 12.5%). In particular, the shrinking behaviour of leaves is a measure of the cell's structural integrity and has a considerable explanatory power for the leaf's hydraulic vulnerability (Scoffoni *et al*. 2014). Hence, we can conclude that in this study, thermal tolerance and hydraulic vulnerability of the species go in the same direction with the more thermal‐tolerant species also being characterized with higher drought tolerance as shown for temperate forest trees (Münchinger *et al*. 2023) and across different other temperate biomes (Mitchell *et al*. 2025). Both studies show a significant correlation between T50 and the leaf turgor loss point (π_tlp_, representing a leaf drought tolerance trait). The species in our study point in the same direction: the more heat‐resistant species, namely Kermes oak and Holm oak, have a π_tlp_ of −3.49 ± 0.14 MPa and −3.26 ± 0.06 MPa, respectively (unpublished data ± SD, measured in Totana, Murcia, Spain). Portuguese oak, which has a higher moisture requirement, has a less negative π_tlp_ of −2.91 MPa (unpublished data, unfortunately without SD values, measured in the Ecological‐Botanical garden in Bayreuth). Despite the low number of species from only one genus sampled in this study, we conclude that the T50 values (both T50_PSII_ and T50_n_) do represent strong physiological traits with a high explanatory power.

## CONCLUSIONS

We could show that the effect of heat duration on leaf thermal thresholds follows predictable patterns. We observed that species differences in thermal sensitivity diminish once the duration of the heat surpasses a certain length. Besides the question of the ecological relevance of long treatments, desired species differences are not present anymore in such long‐lasting heat treatments. Further, heat‐induced necrotic tissue development assessed 2 weeks after the heat treatment does agree well with the temperature‐dependent decline in *F*_v_/*F*_m_. A post‐treatment assessment of necrosis requires a certain degree of leaf xeromorphy to deliver reliable estimates as thin leaves degrade rapidly. Overall, we conclude that T50_PSII_ values are important estimates to predict species responses to heat in comparative species surveys and will greatly assist to detect risks to ecosystems under the aspect of heatwaves accelerating in frequency and intensity.

## AUTHOR CONTRIBUTIONS

NK designed the study. NK and BS collected the data. ED performed the statistical analysis. NK wrote the first version of the manuscript. All authors contributed to drafting the final version of the manuscript.
